# Trends in Robotics Research in Occupational Safety and Health: A Scientometric Analysis and Review

**DOI:** 10.3390/ijerph20105904

**Published:** 2023-05-21

**Authors:** Ci-Jyun Liang, Marvin H. Cheng

**Affiliations:** Division of Safety Research, National Institute for Occupational Safety and Health, Morgantown, WV 26505, USA; mcheng@cdc.gov

**Keywords:** occupational safety and health, robotics, research trends, scientometric analysis

## Abstract

Robots have been deployed in workplaces to assist, work alongside, or collaborate with human workers on various tasks, which introduces new occupational safety and health hazards and requires research efforts to address these issues. This study investigated the research trends for robotic applications in occupational safety and health. The scientometric method was applied to quantitatively analyze the relationships between robotics applications in the literature. The keywords “robot”, “occupational safety and health”, and their variants were used to find relevant articles. A total of 137 relevant articles published during 2012–2022 were collected from the Scopus database for this analysis. Keyword co-occurrence, cluster, bibliographic coupling, and co-citation analyses were conducted using VOSviewer to determine the major research topics, keywords, co-authorship, and key publications. Robot safety, exoskeletons and work-related musculoskeletal disorders, human–robot collaboration, and monitoring were four popular research topics in the field. Finally, research gaps and future research directions were identified based on the analysis results, including additional efforts regarding warehousing, agriculture, mining, and construction robots research; personal protective equipment; and multi-robot collaboration. The major contributions of the study include identifying the current trends in the application of robotics in the occupational safety and health discipline and providing pathways for future research in this discipline.

## 1. Introduction

With the introduction of the Fourth Industrial Revolution (Industry 4.0) concept, new technologies, such as the Internet of Things (IoT), cyber-physical systems, and robotics, have been implemented to increase productivity, improve worker safety, and assist management [1]. The Industry 4.0 concept has been expanded to various industries, including manufacturing [2], warehousing [3], and construction [4], which has raised concerns about occupational safety and health in the workplace. The frequent interaction between human workers and emerging technologies, such as robots, has become a critical challenge in ensuring safety [5].

Recently, the European Commission established a new concept termed the Fifth Industrial Revolution (Industry 5.0) [6]. Compared with Industry 4.0, which is technology-driven, Industry 5.0 focuses on human-centricity, sustainability, and resilience and is value-driven [7]. Creating a safe, friendly, and sustainable workplace using technology is the core of Industry 5.0. Therefore, how the safety of human workers can be ensured, particularly in robotized workplaces, is crucial in the next-generation industry [8].

Robotic technologies are rapidly advancing and being deployed in the workplace. With the growth of sensing, planning, and acting techniques, robots are becoming capable of collaborating with human workers in various tasks in shared workspaces, which is defined as human–robot collaboration or collaborative robots [9,10]. For example, in the manufacturing assembly line, a human and robot can work in the same station and be allocated tasks based on their abilities [11]. In warehouse order picking, several human pickers and robots can travel in a large warehouse to multiple storage locations to pick up and carry orders [12]. In construction sites, human workers and robots can work together on installing building components [13]. Overall, collaborative robots in the workplace typically engage in repetitive, physically demanding, or hazardous tasks, while human workers focus on planning, decision making, or supervising tasks [14,15].

The safety of human workers is important in collaborative robot applications. Previous research has focused on hazard identification, safety system development, risk analysis, and risk management to mitigate robot-related hazards [15,16]. In order to study the current state of collaborative robotics safety, researchers have conducted literature review studies to identify relevant research and suggest future directions. Gualtieri et al. [17] utilized the systematic review method to identify state-of-the-art collaborative robotics research in safety- and ergonomics-related topics. Based on the study results, they presented future research directions, which included contact avoidance, contact detection, and contact mitigation. Robla-Gomez et al. [18] reviewed existing safety systems and regulations for collaborative robots. Arents et al. [19] systematically reviewed collaborative robots in smart manufacturing in terms of worker safety. They suggested that safety mechanisms should be deployed throughout the entire life cycle of collaborative robots. Chemweno et al. [20] reviewed the ISO 15066 standard with reference to collaborative robot safety and the existing research that has studied these safety applications. Villani et al. [21] surveyed state-of-the-art industrial collaborative robot applications regarding worker safety. They determined that the performance of collaborative robots should be considered and optimized in safety system development instead of limiting the performance of robots.

Despite abundant reviews of collaborative robotics safety research, which contribute to the body of knowledge in the field, it is still vital to study the bibliometric relationships between the research on collaborative robots, specifically focusing on the occupational safety and health field, and the impact of the research themes. Compared to a systematic review, a bibliometric analysis or scientometric analysis provides an overview of a specific scientific field, identifies the research trends, and helps determine future research directions. Therefore, this study aimed to analyze the publications, keywords, and citation relationships for the literature in the robotics occupational safety and health field using scientometric analysis [22,23]. The trends and key research themes were identified and further research directions recommended. Through the process of the scientometric analysis, this study attempted to answer the following questions:

1. What are the research trends and sources of publications in the past decade?
2. What are the important keywords in robotics occupational safety and health research?
3. What are the key publications in robotics occupational safety and health research?

## 2. Research Methodology

This study applied the scientometric analysis method to examine existing research on robotics and occupational safety and health. Several keywords were first defined to collect articles from the database. Then, the review process was used to exclude irrelevant articles. Finally, the scientometric analysis was conducted to map robotics in the occupational safety and health research field and identify the trends in the field. The detailed procedure of the study is discussed in the following sections.

### 2.1. Scientometric Analysis

Scientometric analysis is a process of analyzing the quantitative features of the literature in a specific research field [24]. It has been applied in various scientific fields to determine research impacts, indicators, and citation maps, such as in construction [23,25,26], robotics [22,27], and COVID-19 research [28]. Document analysis, including publication year distribution, sources of publications, and co-authorship networks, provides an overview of the research field and the leading researchers. Several citation analysis methods have been used in the past to identify key literature and research groups that impact fields, including keyword co-occurrences, document co-citations, author co-citations, country co-citations, journal co-citations, theme clusters, and citation bursts [29]. Various software tools can be used for scientometric analysis; e.g., VOSviewer, CiteSpace, BibExcel, CoPalRed, Sci2, Gephi, and VantagePoint [22,25]. In this research, we utilized VOSviewer (v1.6.18) [30] and CiteSpace [31] to conduct the scientometric analysis because the functionality provided by the two software tools fit the research objective of network analysis [32].

**Figure 1:**
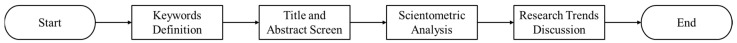
Research procedure.

The research procedure is illustrated in Figure 1. First, keywords were defined to collect literature data from the online database (Scopus). Second, the literature data were screened to exclude irrelevant studies based on the title and abstract. Third, the relevant studies’ bibliographic data were imported and analyzed using VOSviewer. We employed document analysis (years, sources, and co-authorship), keyword analysis (co-occurrences and clusters), bibliographic coupling, and co-citation analysis (authors and documents). Finally, the research trends and future directions are discussed based on the analysis results.

### 2.2. Keywords and Data Collection

In this study, we focused on robotics in occupational safety and health research. The Scopus database was used to collect the literature data since it has a more accurate citation record and has been widely used in past scientometric and bibliometric analyses [22,23,25,28,33]. Web of Science (WOS) is another popular database for scientometric analysis but has less coverage than Scopus. Although some studies combine two databases to conduct scientometric analyses [32,34], we decided to only use the Scopus database in our research.

Keywords need to be clearly defined in the Scopus database to search for related literature. We first defined the keywords “occupational safety and health” combined with several variants—“occupational safety”, “occupational health”, “occupational accident”, and “occupational hazard”—to identify the related research in occupational safety and health. Then, we defined the keywords in the field of robotics. To maximize the collection, we used the term “robot*” to search the literature. The star “*” in “robot*” was used to include every word containing “robot”, such as “robotics”, “robotic”, “robots”, and “co-robot”. The additional keywords we used were “exoskeleton*”, “drone”, “autonomous vehicle”, “UAV” (unmanned aerial vehicle), and “UUV” (unmanned underwater vehicle). Note that the keywords “UGV” (unmanned ground vehicle) and “self-driving car” did not add additional studies, so we excluded these keywords. The final query string applied in the Scopus database using the search field “titles, abstracts, and keywords” was:

(TITLE-ABS-KEY(“occupational safety and health” OR “occupational health” OR “occupational safety“ OR “occupational accident” OR “occupational hazard”) AND TITLE-ABS-KEY(“robot*” OR “exoskeleton*“ OR “drone” OR “autonomous vehicle” OR “UAV” OR “UUV”))

**Figure 2:**
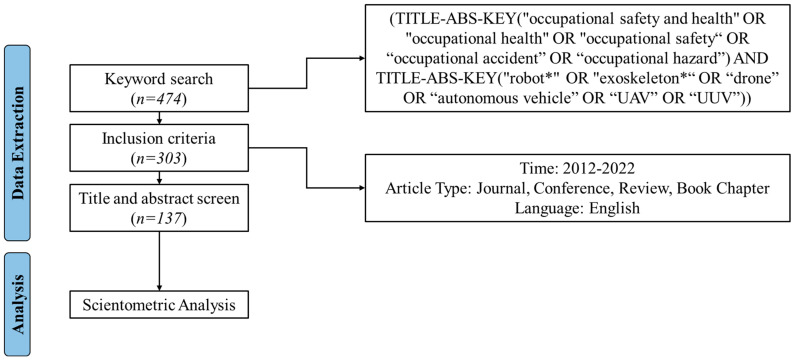
Data extraction and analysis flowchart. The star “*” in “robot*” and “exoskeleton*” was used to include every word containing “robot” and “exoskeleton”.

Figure 2 shows the flowchart for the data extraction and analysis. In the initial search results using the keyword search, we found 474 articles. Note that this search process was conducted on 22 June 2022. To narrow down the search results and only focus on articles published in the past decade, we defined the inclusion criteria as follows:

1. Time: 2012–2022;
2. Article types: journal paper, conference paper, review paper, and book chapter;
3. Language: English.

As a result, we included 303 articles from the Scopus database.

Next, we uploaded the search results to the Covidence website for a further screening process. Covidence is a web-based collaboration software platform designed to streamline the production of systematic and other literature reviews [35]. During this phase, we utilized title and abstract screening to extract relevant articles. Several exclusion criteria were defined to identify the irrelevant articles. First, we excluded articles that were not robotics-related research. Several articles were initially included because the title of the journal or conference contained the word “robot” or they mentioned robots as background or future work. Second, we excluded articles that were not focused on occupational safety and health. For example, several articles focused on using robots to improve productivity and only mentioned occupational safety and health in the background. Third, we excluded articles on surgical, therapy, educational, or military robots. Finally, we excluded some articles for which we were unable to identify the source. After the screening process, a total of 137 articles were included for the scientometric analysis.

## 3. Scientometric Analysis and Results

The scientometric analysis was conducted using document analysis, keyword analysis, bibliographic coupling, citation bursts, and co-citation analysis. The results are discussed in the following sections.

### 3.1. Document Analysis

**Figure 3:**
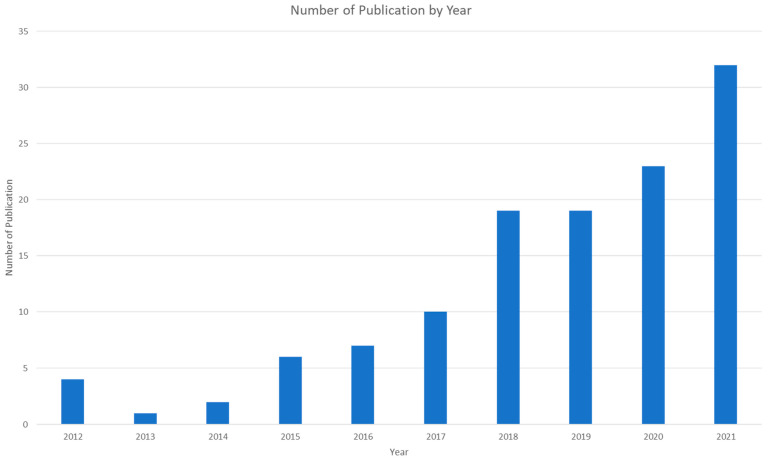
Number of publications by year.

We first analyzed the distribution of articles by year, source of publication, and co-authorship to address the first research question. Figure 3 displays the number of publications by year, showing that, before 2017, there were fewer than ten relevant publications per year. However, after 2017, the number of publications steadily increased to around 20 per year, with a significant jump in 2021 to 32. This trend aligns with the growing interest in human–robot collaboration research [22]. In addition, the COVID-19 pandemic also directed research efforts toward robotics for occupational safety and health [36]. As of 22 June 2022, the number of publications had already reached 14, nearly half the number of publications in 2021.

In terms of the sources of publications, we investigated the number of publications from different journals, conferences, and books. *Applied Ergonomics* and the *International Journal of Environmental Research and Public Health* were the top two journals with the most publications (N = 8) in this field, followed by *Safety Science* (N = 7), which had the greatest number of citations (C = 287). The *American Journal of Industrial Medicine* also had a high number of citations (C = 133). In terms of conference proceedings, *Lecture Notes in Computer Science* and *Procedia Manufacturing* were two notable conference proceedings with high publication (N = 6) and citation numbers (C = 58). Other significant publications included *Advances in Intelligent Systems and Computing* (N = 6), *Robotics and Computer-Integrated Manufacturing* (C = 71), *IEEE Transactions on Automation Science and Engineering* (C = 59), *Information Technology and People* (C = 55), the *Journal of Occupational and Environmental Hygiene* (C = 52), *Ergonomics* (C = 46), *Human Factors and Ergonomics in Manufacturing* (C = 46), and *Sustainability* (C = 34).

**Figure 4:**
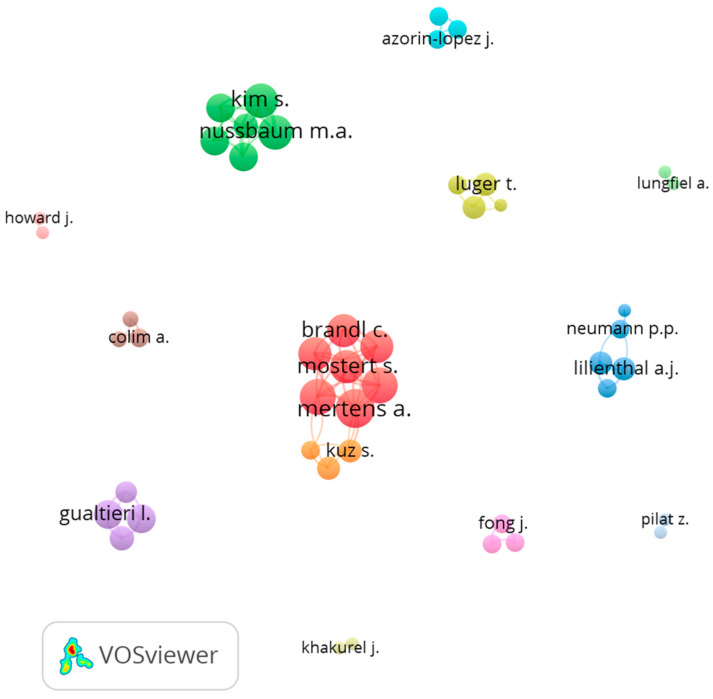
Co-authorship network map [17,38,39,40,41,42,43,44,45].

The co-authorship network analysis was conducted to identify author publication relationships and research collaboration efforts among researchers [37]. This approach can help provide an overview of a research field and identify the leading researchers. Figure 4 illustrates the co-authorship network map in the field of robotics occupational safety and health. The map was developed using VOSviewer co-authorship analysis with authors who had published at least two documents, and there was no citation requirement. As a result, 48 out of 526 authors were included in the analysis. The size of the nodes indicates the weight of the total link strength. There were 12 clusters in the network map, which were sorted by the number of authors in each cluster.

The largest co-authorship cluster (center cluster with red and orange color) comprised a group of researchers from RWTH Aachen University in Germany [38,39]. The second group with a sizeable total link strength (green cluster at the top left of the network map) resulted from collaborative research between Virginia Tech, Rochester Institute of Technology, and King Saud University in Saudi Arabia [40,41,42]. The third cluster (purple cluster at the bottom left of the network map) was a group of researchers from the Free University of Bozen-Bolzano in Italy [17,43]. Finally, the fourth cluster with five researchers (blue cluster at the right side of the network map) was from Örebro University in Sweden [44,45].

The sparse clusters in the map indicate that the research efforts of each research group on robotics in occupational safety and health are limited, since only a few co-authors have published at least two articles. Moreover, among the 12 clusters, only 1 involved cross-university and international collaboration, suggesting that research in this field needs to be expanded to certain regions. Furthermore, most of the collaborative research has been conducted in Europe and North America. Only one publication was from South America and one from the Middle East, with none from Asia, Africa, or Australia.

### 3.2. Keyword Analysis

**Figure 5:**
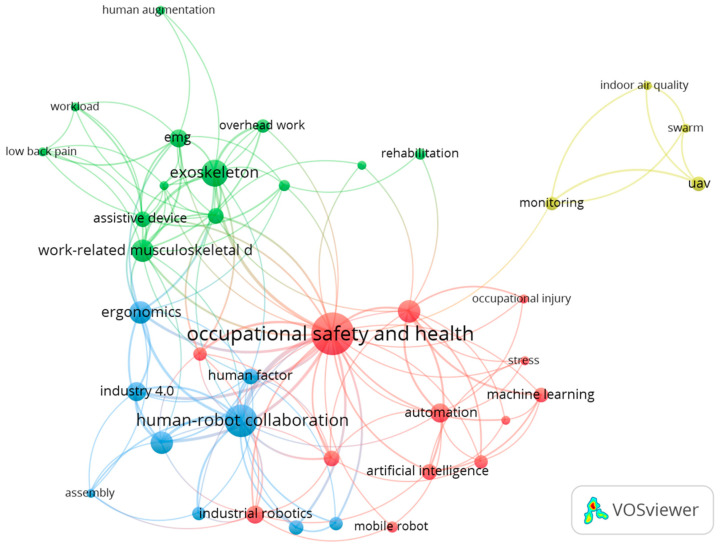
Keyword co-occurrence network map [5,17,40,41,46,47,48,49].

The keyword co-occurrence analysis was conducted to determine the main research areas and important keywords, which were related to the second research question. In order to ensure keyword consistency, the raw author keyword data were first processed to merge similar entities; e.g., “occupational health” and “occupational safety and health”. Next, keywords related to research methodology were ignored; e.g., “systematic review” or “pilot study”. Finally, to determine the important keywords, only keywords with at least two occurrences were included. Figure 5 shows the author keyword co-occurrence network map generated by VOSviewer, utilizing a minimum of two occurrences and a 0.7 layout cluster resolution. Among the 389 author keywords, 39 of them met the keyword requirements. The size of the nodes indicates the occurrence weights and the distance between each node the level of the relationship.

Overall, the top three most commonly occurring keywords were “occupational safety and health”, “human-robot collaboration”, and “exoskeleton”, followed by “collaborative robot”, “ergonomics”, “robot”, and “work-related musculoskeletal disorders (WMSDs)”. Unsurprisingly, the keyword “occupational safety and health” had the most common occurrence since it aligned with the research objective. “Human-robot collaboration” and “collaborative robot” can be seen as similar keywords. However, we decided to separate them since “collaborative robot” focuses on robots that are capable of collaborating with humans [10]. In contrast, “human-robot collaboration” focuses on the collaborative relationship [9]. Exoskeletons, WMSDs, and ergonomics have close relationships and are separate from traumatic occupational injuries. For example, the article by Schmalz et al. [46] contained these three keywords and focused on exoskeletons for overhead work and preventing WMSDs instead of traumatic injuries.

There are four clusters in the network map, each represented by a different color. The first cluster, industrial robot safety, is represented by the red color in the network map. The notable articles in this cluster were those by Badri et al. [5] and Howard [47]. The second cluster represents the keywords “exoskeleton” and “work-related musculoskeletal disorders (WMSDs)” and is shown with the green color in the network map. Most of the articles in this cluster investigated the use of exoskeletons or wearable devices [40,41]. The third cluster represents the keyword “human-robot collaboration” and is shown with the blue color in the network map. This cluster covers human–robot collaboration safety research, such as the articles by Gualtieri et al. [17] and Pearce et al. [48]. The fourth cluster represents the keyword “monitoring” and is shown with the yellow color in the network map. The most cited article in the monitoring cluster was on applying UAVs for construction safety inspection [49].

The keyword “occupational safety and health” had the strongest total link strength and connections with every cluster. The keyword “human-robot collaboration” was second and had connections to industrial robot safety (cluster one) and exoskeletons and WMSDs (cluster two). The keyword “exoskeleton” was the third one with a strong connection to WMSDs. The keyword “ergonomics” lay at the boundary of the human–robot collaboration and the exoskeleton clusters, as some of the articles from the two groups dealt with ergonomics issues. Similarly, the keyword “industrial robotics” belonged to the industrial robot safety cluster but was strongly connected to the human–robot collaboration cluster. Finally, the monitoring cluster did not have many links to the other clusters and only had one link to the “occupational safety and health” keyword. Overall, the scientific interests in this field are occupational safety and health, human–robot collaboration, exoskeletons, and monitoring.

### 3.3. Bibliographic Coupling

**Figure 6:**
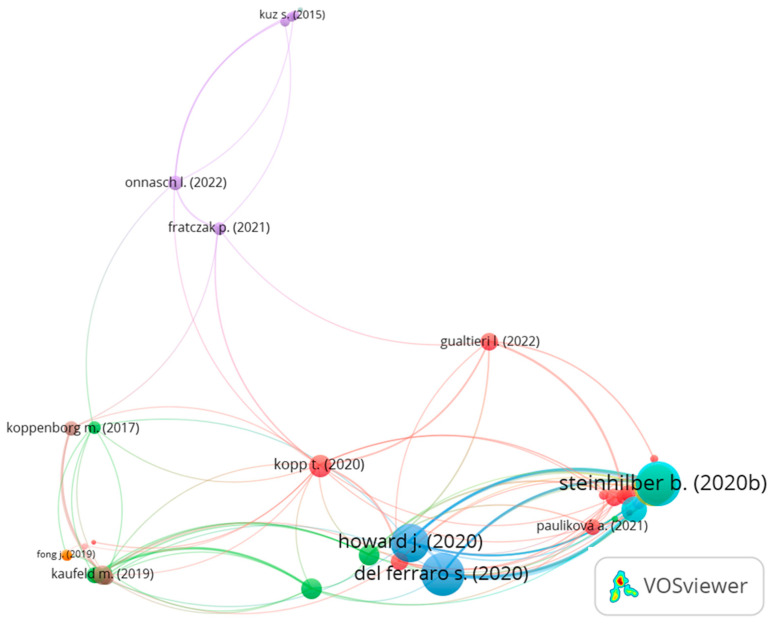
Bibliographic coupling network map [40,41,51,52,53,54,55,56,57,58,59,60,61,62,63,64].

We utilized bibliographic coupling to assess similarities across the literature. Bibliographic coupling refers to two articles citing at least one of the same references [50], and a stronger link between two articles indicates that they both cite more common references. A bibliographic coupling analysis can help identify similar research based on the references rather than keywords. We analyzed bibliographic coupling using VOSviewer and set parameters for at least one citation per article. Among the 137 articles, 102 articles met the criteria. Figure 6 shows the bibliographic coupling network map. Each node represents an article from the analyzed data. The node size represents the total link strength, and the distance between each node represents the reference similarity. Table 1 lists high-link-strength articles from the map.

A group of articles with high link-strength weights at the bottom right side of the map focused on exoskeletons [40,41,42,46,51,52,53,54,55,56,57,58,59,60]. These articles cited similar references on exoskeletons, WMSDs, and ergonomics. The article written by Steinhilber et al. [51] had the highest weight in this group and surveyed the use of exoskeletons to prevent WMSDs. Through a review of the existing scientific literature, the research group developed 20 recommendations and 4 key statements for exoskeletons in relation to WMSD prevention. Del Ferraro et al. [52] systematically reviewed upper-body exoskeletons and their effects on metabolic cost and thermal analysis, highlighting the need for more research effort on the thermophysiological aspect of exoskeletons. Howard et al. [53] discussed the need for intervention research for industrial exoskeletons in terms of analyzing potential benefits and risks, adoption barriers, and potential applications, such as personal protective equipment (PPE).

One of the articles with a high total link strength focused on ergonomics rather than exoskeletons [61] but had similar references to the other exoskeleton articles. This article examined the existing ergonomics and biomechanical risk assessment standards and determined that there is a need to revise these standards by using sensor-based risk assessment methods. Other articles with high total link-strength weights focused on human–robot collaboration [62,63] and the future of work [64]. Kopp et al. [62] investigated the factors for successfully deploying industrial collaborative robots in three phases: decision, implementation, and operation phases. They identified factors related to occupational safety, fear of job loss, trust, robot configurations, financial aspects, and company goals as being critical for the introduction of industrial collaborative robots. Benos et al. [63] studied the ergonomic effects of using UGVs in synergistic agricultural tasks and recommended that the height of the robot be 90 cm. Tamers et al. [64] discussed the perspective of the National Institute for Occupational Safety and Health’s Future of Work Initiative. The priority research topics included organizational design, technological job displacement, work arrangements, AI, robotics, technologies, demographics, economic security, and skills.

### 3.4. Co-Citation Analysis

The co-citation analysis was conducted using VOSviewer and included document co-citation and author co-citation analyses. Co-citation is defined as two articles both being cited by a third article [65]. The link strength between the two articles is higher when they share more citations. In VOSviewer, the co-citation analysis looks at the references of the included articles and finds shared citations, whereas in CiteSpace, the co-citation analysis looks at all the references that cite the included articles. We also analyzed citation bursts using CiteSpace, but no references with significant citation bursts were found. A citation burst is defined as a surge of citations in a period (usually in one year) [66]. A highly cited article has been cited gradually if there is no burst.

**Figure 7:**
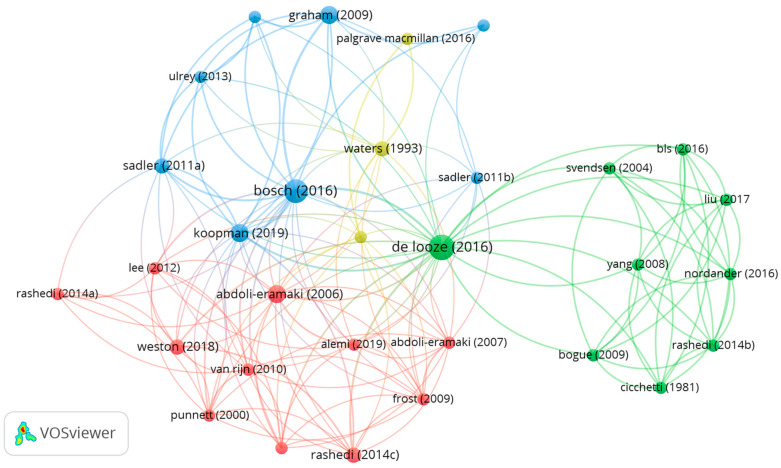
Document co-citation network map [40,41,42,46,51,52,53,54,55,56,57,58,67,68,69,70,71,72,73,74,75,76,77,78,79].

In the documented co-citation analysis, we only included articles with at least two citations, resulting in a total of 31 articles meeting this requirement. Figure 7 displays the documented co-citation network map. Each node indicates an article from the database. The article with the highest total link strength in the center of the map (De Looze et al.) was related to exoskeletons [67] and cited by 18 included articles [40,41,42,46,51,52,53,54,55,56,57,58,68,69,70,71,72,73]. The second article also investigated exoskeletons (Bosch et al. [74], blue node) and was cited by 11 articles [41,42,51,53,54,55,57,58,72,73,75]. The third article investigated exoskeletons for low-back loading (Koopman et al. [76], blue node connecting to the red cluster) and was cited by four articles [51,56,57,58]. Overall, the references with high link strengths in our data were focused on WMSDs and ergonomics research using exoskeletons [67,74,76,77] or lift-assist devices [78,79].

**Figure 8:**
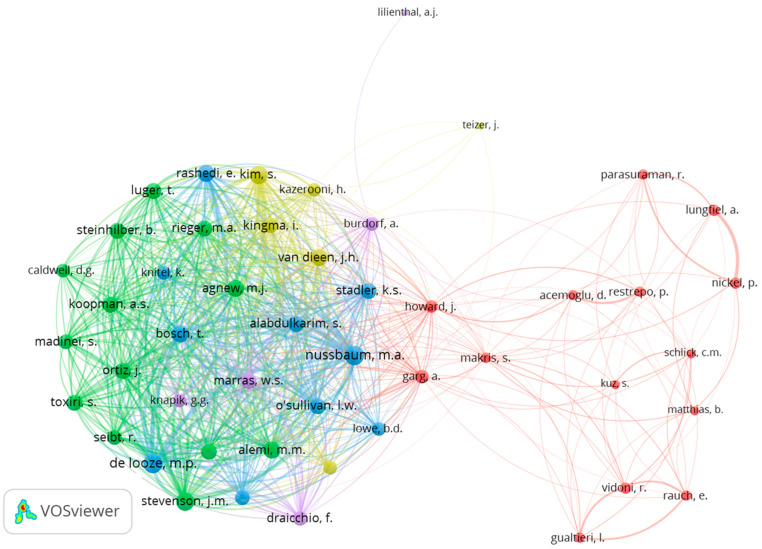
Author co-citation network map.

For the author co-citation analysis, we set the threshold to authors with at least 12 citations, which resulted in 48 authors meeting the threshold. Figure 8 shows the author co-citation network map. The author with the highest link strength (Nussbaum, M. A.) and the third, fifth, sixth, and seventh authors in rank (Kim, S., Bosch, T., Alemi, M. M., and Agnew, M. J.) co-authored articles on exoskeletons with high co-citation numbers [40,41,42,55,60]. The second author in rank (de Looze, M. P.) published ten articles on exoskeleton topics [67,74,76,80,81,82,83,84,85,86]. The fourth author (Stevenson, J. M.) had publications related to personal lifting assistance/augmentation devices and how they can improve human work-related ergonomics [78,79,87,88,89,90,91,92].

On the right side of the map, there is a different author cluster that does not have a close relationship to the abovementioned authors. The two authors with high link strengths are in the middle of the map (Howard, J., and Garg, A.) and connect to the exoskeletons and ergonomics author groups since they have written articles not only on exoskeletons [53] and ergonomics [93] but also on robotics and AI [47,94,95]. At the top of the map, there are two authors who do not have much connection to the other authors. One of them has focused on construction automation [96] (Teizer, J.), while the other has focused on air quality monitoring using mobile robots and sensor networks [44,45,97] (Lilienthal, A. J.).

## 4. Discussion

This section discussed each keyword cluster and major publications to identify research trends or gaps in robotics and occupational safety and health. We then recommend future research directions based on these trends and gaps.

### 4.1. Research Trends

To answer the first research question on research trends in the past decade, keyword clusters were defined in Section 3.2. Four clusters were identified: industrial robot safety, exoskeletons and work-related musculoskeletal disorders (WMSDs), human–robot collaboration, and monitoring. Table 2 lists all keywords and their corresponding clusters, along with the average numbers of citations and the occurrences of each keyword, to determine high-influence keywords. The key publications in each cluster are also discussed (third research question); i.e., articles with at least ten citations.

In the industrial robot safety cluster, the most cited article was written by Badri et al. [5], which investigated the integration of Industry 4.0 and occupational safety and health. It is important to ensure a smooth transition in Industry 4.0 in order to avoid a negative impact on occupational safety and health. Murashov et al. [95], Howard [47], and Tamers et al. [64] discussed collaborative robots and AI in the future workplace and provided recommendations for occupational robot safety, such as the development of standards, the establishment of risk profiles for robotized workplaces, and redundant safety system development. Other AI-related issues in the future workplace, such as job displacement and safety management, were also pointed out. Leso et al. [98] reviewed the literature on Industry 4.0 and the occupational safety and health of workers. Although the deployment of robots can make the workplace safer, psychological stress and additional safety risks can be introduced to workers. Risk assessment and management are required to mitigate occupational hazards. James et al. [99] studied the impact of automation on the mental workload of pharmacy staff, indicating that it could improve working conditions and workload. Brocal et al. [100] proposed a method (TICHNER) to identify the new and emerging risks associated with the automatic manufacturing process. Paulikova et al. [101] used the strength, weakness, opportunities, and threats (SWOT) methodology to analyze the impact of robotized manufacturing workplaces with regard to occupational safety and health. The impact on human workers’ well-being is an important aspect that has not been fully studied.

In the exoskeletons and WMSDs cluster, the majority of the research focused on assessing exoskeletons for different industrial tasks, such as overhead work [40,41,42,46], demonstrating the benefits of using exoskeletons to reduce workload and WMSDs [53]. Khakurel et al. [68,69], Del Ferraro et al. [52], and Bär et al. [56] reviewed wearable technologies in the workplace, including wearable robots and exoskeletons, and identified their benefits in terms of workers’ well-being and reducing work-related injuries and WMSDs. Baltrusch et al. [58] evaluated the use of exoskeletons in repetitive lifting and walking tasks. The results indicated that exoskeletons could reduce the metabolic costs for human workers in lifting tasks, but these increased in walking tasks. Cho et al. [102] tested exoskeletons for construction lifting tasks in order to maintain an acceptable posture for construction workers. The experiment results indicated that the use of exoskeletons can keep masons in appropriate postures when lifting heavy concrete masonry units. Sado et al. [70] developed an exoskeleton control method based on the dual unscented Kalman filter and the impedance supervisory controller to synchronize the exoskeleton’s movement with the human’s walking movement, which yielded an over 40% reduction in lower limb muscle activity in load-carrying scenarios.

In the human–robot collaboration cluster, research objectives were diversified. Ergonomics safety was one of the popular topics in human–robot collaboration research [17,103,104]. Pearce et al. [48] developed a task allocation approach to deploy collaborative robots to manufacturing processes by optimizing the makespan (i.e., task completion time) and human physical strain. Three factors (completion time, total strain, and work idle time) were identified and the authors discussed how to balance these factors in the schedule optimization process. Faber et al. [38] analyzed human–robot shared ergonomic workspace design requirements for assembly lines. The design of collaborative assembly workstations has demonstrated improved ergonomics and productivity [43,105]. Guo et al. [106] evaluated a robotic hospital patient transfer system (i.e., robotic bed movers) in terms of the human physical strain and how it can improve the health of hospital workers; a reduction in back muscle activities was shown in the experiment. The human–robot relationship is also important in ensuring safety. Koppenborg et al. [107] compared the effects of the speed and path predictability of an industrial collaborative robot on the human collaborator. Kopp et al. [62] studied the factors for successfully deploying collaborative robots in industry. The results indicated that it is not only technology-related factors that influence the deployment outcome but employee factors, such as job replacement, trust relationships, and acceptance, also play significant roles. Rebuilding a trust relationship between humans and robots after an incident is one way to ensure human workers’ mental well-being. For example, the robot could apologize to the human collaborator if it made a mistake using pre-recorded audio (e.g., “Sorry for hitting you.”) [108].

In the monitoring cluster, most articles focused on safety and air quality monitoring. For example, Melo et al. [49] examined the feasibility of using UAVs for construction safety inspections based on two case studies. The benefits and barriers of the UAV safety inspection were compared. Howard et al. [94] reviewed UAV applications in construction, including monitoring, inspection, maintenance, and construction work, and highlighted the importance of safety in this field. Bennetts et al. [44,45] utilized mobile robots, localization methods, and sensor networks to monitor air quality in the industrial workplace. Their system leverages the spatial ability of mobile robots and the temporal ability of sensor networks to collect air quality data. Pagano et al. [109] proposed an optimal design approach for structure-inspection inchworm climbing robots to address environmental, mobility, and safety factors. Munoz Martinez et al. [110] developed a mobile robot, MineBot, to monitor chemical, physical, and biological particles within mines. The MineBot is equipped with a teleoperating system, allowing human operators to control it remotely.

### 4.2. Future Directions

Based on the research trends in robotics occupational safety and health in the past decade, several emerging research directions are identified and discussed for researchers and interested industrial collaborators. First, according to the scientometric analysis results, most of the research in this area has focused on the manufacturing industry. Many robotics applications in different industries have yet to be intensively studied regarding occupational safety and health, such as warehouse, agriculture, mining, and construction robots. For the warehouse workplace, human workers and autonomous mobile robots (AMRs) navigate a shared workspace, and potential hazards can arise. It is important to assess the risk [111] and detect potential hazards in such scenarios [112]. Furthermore, measuring the mental workload of the human collaborator is a factor in minimizing risk when working with warehouse robots [113]. Similarly, in agriculture, robots have to work with human workers in outdoor and muddy environments, and it is a challenge for them to collaborate in such workplaces, which can result in unique safety hazards. How to alleviate occupational injuries is a critical consideration for agriculture robotics research [114,115]. For example, adopting collaborative robot safety standards in the agriculture workplace to ensure the safety of human workers could be one approach [115].

The mining and construction industries are also hazardous working environments where robots can help reduce the workload of human workers. However, it is difficult for robots to work in unstructured and dynamic environments. Human–robot collaboration is necessary to overcome these challenges. The safety concerns regarding human–robot collaboration in these industries should be a priority. Although existing research has examined this topic using sensors, the Internet of Things (IoT), and computer vision methods [116,117,118], the research field is still open-ended and needs more effort. Moreover, the development of collaborative robot safety standards specific to mining and construction robots is essential. The collaborative robot safety standards published by the Robotic Industries Association (RIA) [119,120] and International Organization for Standardization (ISO) [121,122,123] target industrial environments and require additional modifications for application to mining and construction environments. Finally, the industry adoption of construction robots is still limited because of the cost and technology barriers [124]. Human worker acceptance is another factor that delays robot adoption at construction sites [125,126]. Research on improving construction robot acceptance is needed.

Second, the design of and requirements for personal protective equipment (PPE) for those working with collaborative robots need to be considered. In traditional robot workstations, the human and robot are separated in different workspaces to prevent hazards. In collaborative robot workstations, in contrast, the human and robot share the workspace or even make contact with each other sometimes. Even though the standards have strict safety requirements for collaborative robots, unexpected contact might occur and cause serious injuries. One way to reduce this hazard is to determine the speed limit for the collaborative robot that would keep the impact-transferred energy under acceptable thresholds. The permissible impact forces and deformation of the human body determined by the safety standard [123] should be considered when setting the maximum speed of the robot. Another way to protect human workers is to wear proper PPE. At typical manufacturing or construction sites, wearing PPE, such as a helmet and safety glasses, is mandatory. When collaborating with robots, additional PPE should be considered or designed since the robot might impact the human from various directions in the configuration space.

Third, human and multi-robot collaboration is garnering attention in the industry as a way to increase efficiency and balance costs. Human workers can work with multiple mobile robots in the warehouse to gather and transport goods [127]. In the construction industry, the combination of a UAV and mobile robot can be used to collect more reliable environmental data [128,129], or multiple affordable robots can be used to detect fall hazards [130]. As more collaborative multi-robots are deployed in the workplace, it will become necessary to study the impact on human workers. For example, determination of the trust or comfort level of human workers when collaborating with multiple robots and continuous tracking of all entities in the workspace using sensors, computer vision, and the IoT will be needed. Information systems, such as digital twins or building information modeling (BIM), can be useful for monitoring real-time activities in the complex workplace [14,131,132]. Multi-robot deployment and allocation is another research direction in occupational safety and health. Collision avoidance for multi-robot systems should be investigated to ensure the safety of human collaborators [133,134]. In addition, task allocation for multi-robot systems and human workers can allow humans to build trust in the robot system and achieve a safe working environment [135,136].

## 5. Conclusions

This research investigated the scientometric relationships in the field of robotics occupational safety and health over the past decade. The study employed co-authorship analysis, bibliographic coupling, co-citation analysis, and keyword analysis to answer three research questions and identify key publications in the field. The contributions to the body of knowledge included determining the research trends and analyzing citation relationships in this field. The four identified research trends were industrial robot safety, exoskeletons and work-related musculoskeletal disorders (WMSDs), human–robot collaboration, and monitoring. The corresponding key publications were reviewed and discussed. Based on the findings, future research directions were recommended and included occupational safety and health research on warehousing, agriculture, mining, and construction robots; PPE for workers near collaborative robots; and multi-robot collaboration.

The limitations of the research are threefold. Firstly, the literature collection was highly dependent on the definition of the search keywords. For example, although the search keyword included “autonomous vehicle”, there were no relevant articles included in the results. As autonomous vehicles are emerging in the occupational transportation sector, future scientometric analyses should also investigate this direction. Secondly, the search results may vary depending on the database used. Our study utilized Scopus to collect articles, but the search results may differ in the Web of Science and Google Scholar databases. In future research, multiple databases should be used to collect data. Thirdly, citation analysis was the key factor in this study. However, using citations to evaluate the impact of research publications remains controversial. Researchers argue that, with only citation analysis, the impact and quality of the literature cannot be fully estimated [25]. Additional indicators should be considered in future research to interpret the findings.

## Figures and Tables

**Table 1:** Articles with high total link strength in the bibliographic coupling network map.

Article	Links	Total Link Strength	Citations
Steinhilber et al. [51]	19	90	5
Del Ferraro et al. [52]	20	80	15
Howard et al. [53]	19	66	24
Steinhilber et al. [54]	22	61	7
Park et al. [55]	17	60	1
Bär et al. [56]	17	59	9
Schwartz et al. [57]	17	53	1
Ranavolo et al. [61]	21	51	8
Alabdulkarim et al. [42]	15	50	18
Baltrusch et al. [58]	17	45	46
Kim et al. [41]	15	44	75
Schmalz et al. [46]	15	43	37
Kim et al. [40]	12	29	87
Zelik et al. [59]	13	29	2
Schwerha et al. [60]	16	28	2
Kopp et al. [62]	16	23	9
Benos et al. [63]	14	21	1
Tamers et al. [64]	10	19	38

**Table 2:** Cluster, keywords, number of occurrences, and mean citations.

Cluster	Keywords	Occurrences	Mean Citations
Industrial robot safety	Occupational safety and health	40	16
	Robot	11	7
	Automation	8	5
	Industrial robotics	7	10
	AI	6	10
	Risk assessment	6	13
	Machine learning	5	13
	Manufacturing	4	4
	Workplace	4	33
	Mobile robot	3	4
	Future of work	2	21
	Occupational injury	2	3
	Stress	2	14
Exoskeleton and WMSDs	Exoskeleton	16	22
	WMSDs	11	12
	EMG	7	14
	Assistive device	6	18
	Wearable technology	6	16
	Overhead work	4	44
	Biomechanics	3	15
	Rehabilitation	3	1
	Firefighter	2	1
	Human augmentation	2	13
	Low back pain	2	24
	Oxygen consumption	2	31
	Workload	2	3
Human–robot collaboration	Human–robot collaboration	23	12
	Collaborative robot	11	18
	Ergonomics	11	17
	Industry 4.0	8	35
	Human factor	6	14
	Virtual reality	5	12
	Anthropomorphism	4	2
	Human–machine interaction	4	3
	Assembly	2	18
Monitoring	UAV	5	28
	Monitoring	4	5
	Indoor air quality	2	3
	Swarm	2	3

## Data Availability

The data presented in this study are available on request from the corresponding author.

## Disclaimer

The findings and conclusions in this report are those of the authors and do not necessarily represent the official position of the National Institute for Occupational Safety and Health, Centers for Disease Control and Prevention. Mention of any company or product does not constitute endorsement by the National Institute for Occupational Safety and Health, Centers for Disease Control and Prevention. In addition, citations to websites external to the NIOSH do not constitute NIOSH endorsement of the sponsoring organizations or their programs or products. Furthermore, the NIOSH is not responsible for the content of these websites. All web addresses referenced in this document were accessible as of the publication date.
